# Androgen Receptor (AR), E-Cadherin, and Ki-67 as Emerging Targets and Novel Prognostic Markers in Triple-Negative Breast Cancer (TNBC) Patients

**DOI:** 10.1371/journal.pone.0128368

**Published:** 2015-06-03

**Authors:** Giuseppina Rosaria Rita Ricciardi, Barbara Adamo, Antonio Ieni, Luana Licata, Roberta Cardia, Giuseppa Ferraro, Tindara Franchina, Giovanni Tuccari, Vincenzo Adamo

**Affiliations:** 1 Medical Oncology Unit AOOR Papardo-Piemonte & Department of Human Pathology University of Messina, Messina, Italy; 2 Oncologia Mèdica Hospital Clínic, Barcelona, Spain; 3 Department of Human Pathology “Gaetano Barresi”, Section of Anatomic Pathology, University of Messina, Messina, Italy; University Campus Bio-Medico, ITALY

## Abstract

**Background:**

TNBC is an aggressive subset of breast cancer (BC) without specific target therapy.

**Methods:**

This observational, retrospective study included 45 cases of TNBC. The aim of this study was to evaluate the expression of the AR, E-cadherin and Ki-67 in relation to histological type, time to relapse and overall survival (OS). Immunohistochemistry (IHC) was carried out on formalin-fixed paraffin-embedded tumor samples obtained from patients defined TNBC.

**Results:**

The AR was positive (IHC >10%) in 26.6%. E-cadherin (CDH1) expression was considered positive if the score was ≥ 2. This expression was negative in 53.3% cases. The Ki-67 index was ≥ 20% in 37.7%. Univariate analyses showed that AR, CDH1 and Ki-67 are significantly associated with OS. Multivariate analysis showed that AR and Ki-67 expression are independent variables associated with OS. The statistical analysis showed that patients with AR negative and Ki-67 positive expression have a significant correlation with poor outcome.

**Conclusions:**

Our data suggest that the combination of AR and E-cadherin expression as well as Ki-67 status might be useful prognostic markers in TNBC. Hence, these molecular determinants could play an interesting role to classify subgroups of TNBC.

## Introduction

Breast cancer is a heterogeneous disease with different morphologies, molecular profiles, clinical behavior, response to therapy and patient outcomes [1].

Triple-negative breast cancer (TNBC) represents approximately 15% of all breast cancers and is a subtype distinguished by lack of expression of the estrogen and progesterone receptor by immunohistochemistry (IHC) and by the lack of overexpression and/or amplification of HER2 obtained with IHC and or fluorescence in situ hybridization (FISH). TNBC do not benefit from endocrine therapy or therapies targeted to human epidermal growth factor receptor type 2 (HER2) in contrast with the other subtypes [2–5].

TNBC is diagnosed more frequently in younger patients, with BRCA1 mutations and in premenopausal and African-American women. Compared to the other subgroups of tumors, TNBC is biologically more aggressive and is associated with higher recurrence rates during the first 1–3 years and higher frequency of metastatization to visceral organs and central nervous system (CNS) with lower rates of bone disease and poor overall survival in the five years after diagnosis [6–8].

The clinic-pathological characteristics of this subtype include tumors of large size, highly undifferentiated, high proliferative index, central necrosis, multiple apoptotic cells and high positive lymph nodes. The predominant histological type is ductal and less frequently the others, metaplastic and medullary [9].

However, triple negative breast cancer is a heterogeneous disease since it includes different molecular subtypes, such as the basal-like subtype and claudin-low [10].

Although there are numerous similarities between basal-like and triple-negative breast cancers and some have previously used these terms interchangeably, they are not synonymous [11]. Indeed, it is true that the majority of triple negative cancers have basal-like phenotype and the majority of tumors expressing ‘basal’ markers are triple-negative [12–15]. Thus, only 71% of triple negative tumors are basal-like by gene profiling expression, and only 77% of basal-like tumors are triple negative [14,16]. Is interesting to note that TN tumors that do not express a basal-like phenotype may have a better prognosis than TN basal-like tumors [17].

Moreover, an additional BC subtype, named as claudin-low and partly overlapping with the IHC-defined TNBC, has been recently identified. It is characterized by low expression of claudin genes, which are important for cell-cell adhesion, and often presents with stem-cell and epithelial-to-mesenchymal transition features [10,18].

Lehmann et al. by a recent gene analysis expression of TNBC identified at least 6 different tumor molecular subtypes including two basal-like (BL1 and BL2), an immunomodulatory (IM), a mesenchymal (M), a mesenchymal stem-like (MSL), and a luminal androgen receptor (LAR) subtypes, which appear to be driven by distinct pathways that may be effectively targeted by specific drugs in *vitro* [19]. In particular, Lehmann et al. investigated the molecular features of the AR + TNBC subtype, showing that activating PIK3CA mutations are enriched in AR + TNBC and this provide rationale for investigate the use of AR antagonists in combination with PI3K/mTOR inhibitors in this specific subtype [20].

Burstein et al., using RNA and DNA genomic profiling have defined 4 clinically- relevant subtypes of TNBC characterized by distinct clinical outcomes and molecular signatures defined by specific over-expressed or amplified genes molecular signatures providing the basis for molecularly-targeted and/or immune-based strategies in these aggressive tumors [21].

Recently new panel of biomarkers were identified in order to provide both prognostic and predictive information in TNBC. Among them, some of the most promising markers are the Androgen receptor (AR), E-Cadherin and Ki-67 expression.

AR, a member of the steroid hormone receptor family, is expressed in more than 70% of breast cancers and has been implicated in breast cancer pathogenesis [22, 23].

The gene for the AR is located on chromosome Xq 11–12. The androgen receptor is composed by a single polypeptide with four domains with different functions. After ligand binding (endogenous androgens or other growth factors), the AR that is usually bound to chaperone proteins, such as heat shock proteins, it dissociates from these proteins and forms a homodimer that translocates to the nucleus and leading to a signaling cascade that results in the activation of target genes transcription. Despite the prevalence of AR expression in both normal breast tissue and primary tumors, its clinical role in breast cancer is less well known [24–26].

Preclinical studies have shown that androgens can cause a proliferative change in the cell lines affected by breast cancer and to promote tumorigenesis. In addition, the high concentration of 5α-dihydrotestosterone stimulates the proliferation of breast cancer cell lines MCF-7 and EFM-19 estrogen-responsive [27].

However, the stimulatory effect of androgens on the proliferation of breast cancer is not just limited to cell lines ER positive. The proliferation induced by androgens has also been demonstrated in breast cancer cell lines (MDA-MB-453) AR, ER and PgR negative when incubated with the synthetic, not metabolized androgen, Mibolerone [28]. This suggests a connection between androgens and breast carcinogenesis, and AR may be useful as a therapeutic target for triple-negative breast cancers. However, recent retrospective studies suggested that AR status was a significant prognostic marker in TNBC [29–31].

The E-Cadherin is a cell adhesion molecule, synthesized by the CDH1 gene located in chromosome 16q22.1, expressed on all epithelial cells, that acts as cell proliferation, invasion and a metastasis suppressor. E-cadherins have a transmembrane glycoprotein structure with three distinctive domains: intracytoplasmic, transmembrane, and extracellular, respectively. The cytoplasmic domain performs a double role, of junctional/structural proteins and of signal transducer [32]. The extracellular domain (EC) is composed of four subdomains: EC-1 (calcium-dependent), EC-2, EC-3, EC-4 (adherent subdomains) adjacent to the membrane proximal extracellular domain [33]. E-cadherin has a key role in the cellular motility and invasion during the epithelial–mesenchymal transition process and in extravasation and migration during the mesenchymal–epithelial one [34].

In the majority of epithelial tumors, the function of E-cadherin is lost by inactivating mutations of the genes encoding E-cadherin and β-catenin, inhibition of transcription or proteolysis of the extracellular domain of cadherin. Experimental evidences indicate that the E-cadherin expression loss is crucial for the acquisition of the invasive and metastatic capacities of the epithelial tumors [35]. Retrospective studies have shown that a low-level expression of E-cadherin is a marker of poor prognosis [36, 37].

Ki-67 is a non-histone nuclear protein associated with cellular proliferation and originally identified by Gerdes and collegues in the early 1980s. The cellular location of this protein varies throughout the cell cycle, with different detectable levels during G1, S, G2 and M phases and not during the resting phase G0. Its precise function is uncertain, but the tight association with cell-cycle phase regulation and its short half-life render this protein a valid marker for the assessment of the growth fraction of neoplastic cell populations. The use of Ki-67 immunostaining as a prognostic and predictive marker in breast cancer has been extensively evaluated, although there is no standard cut-off definition yet [38–40].

The aim of this study is to discover additional prognostic markers that can better classify TNBC and identify tumors with more aggressive behavior. For this purpose, we investigated the expression of various molecular determinants such as AR, E-Cadherin and Ki 67 in TNBC, and explored their correlations with morphological characteristics as well as the outcome of TNBC.

## Patients and Methods

This observational, retrospective study included 45 consecutive cases of TNBC from 2001 to 2011.

Patients were eligible for the study if they met the following criteria: women aged ≥18 years; histological diagnosis of breast cancer (stage I–IV according to TNM [tumor, node, metastasis] American Joint Committee on Cancer [AJCC] version VI) availability of the following local staging and biological parameters: pT, pN, grade (G). All patients were defined TNBC, since ER and PgR cell stainings of both were 0% by IHC, and HER2 staining of 0 by IHC or 1+ and 2+ score with no gene amplification verified by fluorescence in situ hybridization (FISH).

Immunohistochemistry (IHC) was carried out on formalin-fixed paraffin-embedded tumor samples for the determination of androgen receptor, E-cadherin and Ki-67.

Tissue sections were then incubated with each primary monoclonal antibody against AR (clone AR 411, dilution 1:100), E-Cadherin (clone NCH-38, dilution 1:200), Ki-67 (clone MIB-1, dilution 1:100).

Sections were considered AR positive when ≥10% of tumor cell nuclei stained positive [36, 41]. E-cadherin expression was semi-quantitatively analyzed according to the percentage of cells showing membrane positivity: 0 (0 to 10%); 1+ (10 to 30%); 2+ (30 to 70%); 3+ (>70%) [35, 36]. E-cadherin expression was considered positive if the score was ≥ 2, and negative when score was ≤ 1.

We considered a high Ki67 index > 20% [42].

This retrospective, non interventional study was conducted on archived tumor sections, for whom informed written consent was obtained from donors or the next of kin for the use of tumors sample in research. We did not consult an Institutional Review Board, considering the retrospective nature of our study. In our Center, being an Academic Institution, we usually collect written informed consent of all patients, after the histologic confirmation of cancer, in order to perform no profit research projects. Given the characteristics of this study, a representative of the Comitato Etico Interaziendale della Provincia di Messina confimed that this work did not require ethical approval, but it was necessary only an informed written consent.

## Statistical Analysis

Associations between androgen receptor-negative/Ki-67 expression and either ductal type or histological grade 3 were determined using Chi-square or Fisher’s exact test. In addition, we examined the association between the various clinical-pathological variables (i.e. androgen receptor status, histological type, histological grade, Ki-67 expression and E-cadherin expression) and overall survival. Survival curves were plotted using the Kaplan-Meier method and differences between the survival curves were determined using the log-rank test. Univariate and multivariable analyses were performed using Cox proportional hazard models. A two-sided p-value of <0.05 was considered significant. All analyses were performed using R package 2.15.1 (www.r-project.org).

## Results

**Fig 1:**
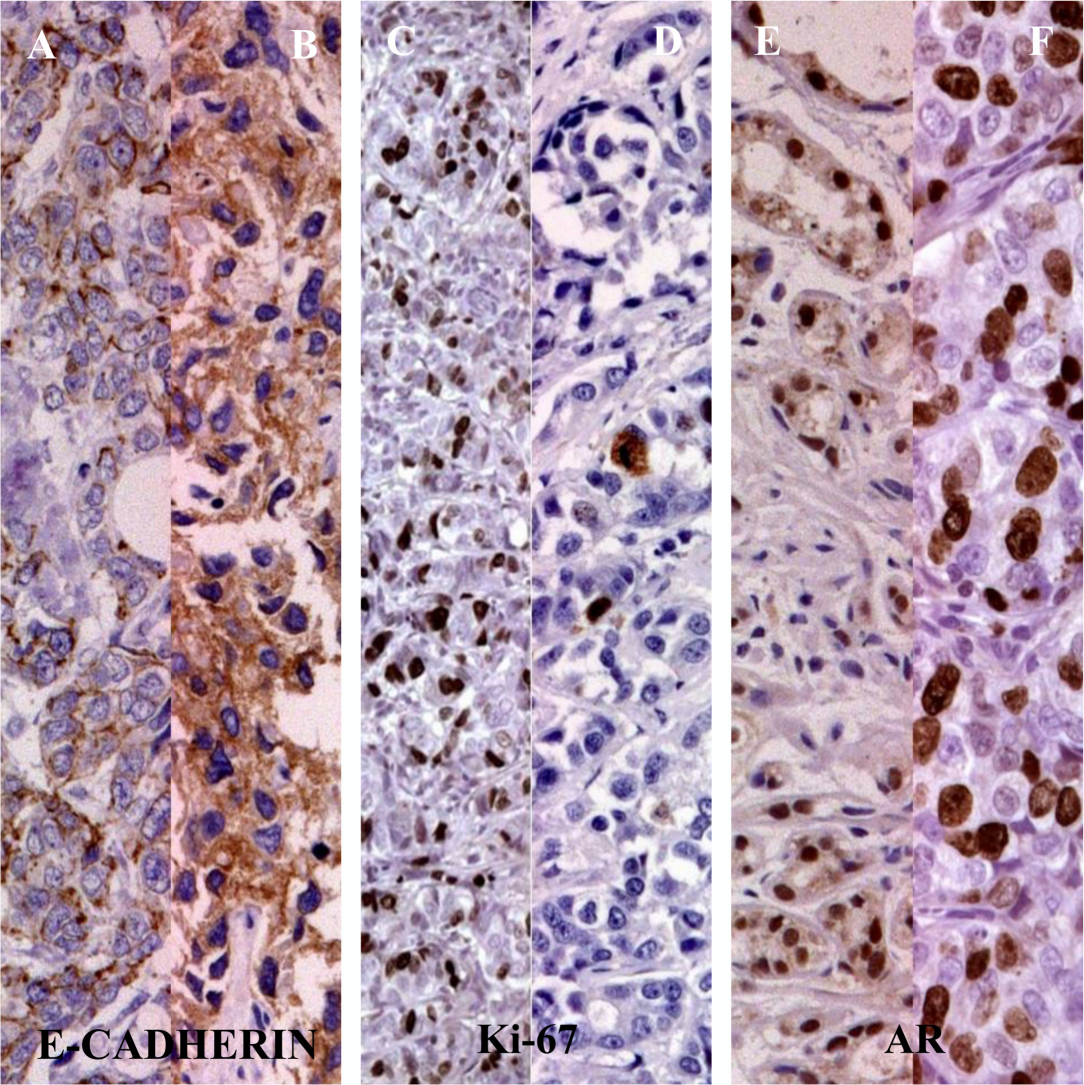
The AR, E-Cadherin and Ki 67 expression in 45 patients TNBC. Legend: [A, B] negative/positive AR staining; [C, D] E-cadherin negative/positive staining; [E, F] Ki-67 level < or ≥ 20%.

We have analyzed in 45 consecutive patients TNBC the androgen receptor, E-cadherin and Ki-67 expression (Table 1 and Fig 1).

**Table 1:** AR, E-Cadherin and Ki 67 expression.

Molecular Determinants	N (%)
**AR**	
Positive	12/45 (26.6%)
Negative	33/45 (73.3%)
**E-cadherin**	
Positive	24/45 (53.3%)
Negative	21/45 (46.6%)
**Ki 67 index**	
≥20%	17/45 (37.7%)

Patient and tumor characteristics are summarized in Table 2.

**Table 2:** Characteristic of patients.

Variable	N (%)
**Total**	45
**Median age**	58.8 years (range 39–77)
**Histological Type**	
Ductal	35 (77.7%)
Lobular	7 (15.5%)
Medullary	3 (6.6%)
**Grade**	
G2	16 (35.5%)
G3	29 (64.4%)
**Tumor Stage**	
I	6/45 (13.3%)
II A	21/45 (46.6%)
III A	11/45 (24.4%)
III B	3/45 (6.6%)
IV	4/45 (8.8%)

**Fig 2:**
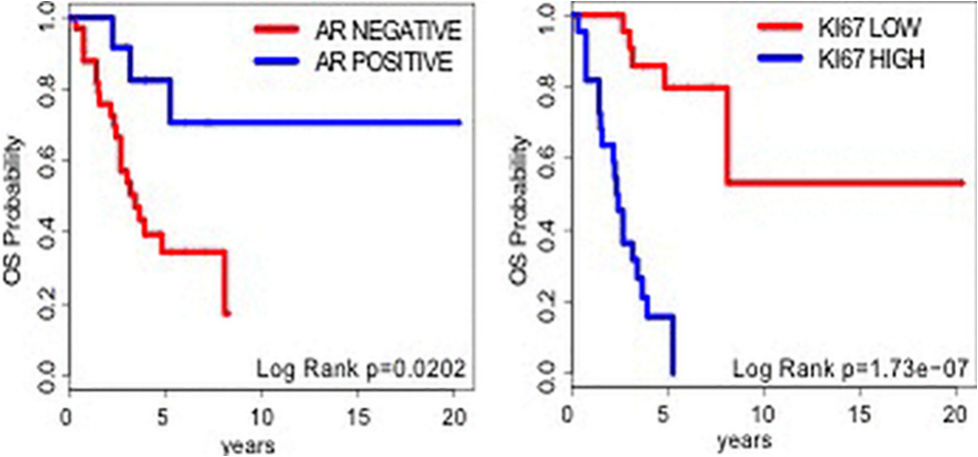
Estimated Overall Survival Curves for AR and Ki67. The AR and Ki-67 expression are independent variables associated with overall survival.

Median age was 58.8 years (range 39–77). The main histological type was ductal in 35 pts (77.7%), lobular in 7 (15.5%), medullary in 3 (6.6%). 29 pts (64.4%) had a G3 tumor. Tumor stage was: I 6/45 (13.3%), IIA 21/45 (46.6%), IIIA 11/45 (24.4%), IIIB 3/45 (6.6%) and IV 4/45 (8.8%) (Table 2). All patients received treatments; the most frequently used regimens were anthracycline and taxanes. The androgen receptor was positive (IHC >10%) in 12/45 (26.6%). E-Cadherin (CDH1) expression was negative in 24/45 (53.3%). The Ki-67 index was ≥ 20% in 17/45 (37.7%). Univariate analyses showed that AR, CDH1 and Ki-67 are significantly associated with overall survival. Multivariate analysis showed that AR and Ki-67 expression are independent variables associated with overall survival (Table 3 and Fig 2).

**Table 3:** Univariate and multivariate analysis.

Table 3. UNI/MVA	Univariable cox model				Multivariable cox model			
Variable	Hazard Ratio	95% INF	95% SUP	p-Value	Hazard Ratio	95% INF	95% SUP	p-Value
**Grade G3 vs G2**	1.54	0.65	3.62	0.32	-	-	-	-
**AR+ vs AR-**	0.26	0.08	0.88	0.03	0.15	0.04	0.59	0.01
**CDH1+ vs CDH1 -**	0.33	0.14	0.80	0.01	0.48	0.18	1.29	0.15
**Age (var. cont)**	1.00	0.97	1.03	0.97	-	-	-	-
**Ki 67 (var. cont)**	26.70	5.95	1119.70	<0.0001	48.28	6.57	354.65	0.0001

The Kaplan-Meier curves showed that patients with AR negative and Ki-67 positive expression have a significant correlation with poor outcome (Fig 2).

## Discussion

Triple negative breast cancer is an aggressive subset and represents a molecular subtype without specific target therapy [1, 18, 43, 44].

Moreover, TNBC is a heterogeneous disease and the identification of the molecular prognostic markers is necessary to allow a better characterization of this subtype and for the construction of appropriate therapeutic strategies. Several studies shown that in ER-negative tumors, AR expression has been associated with significantly better disease-free survival than AR-negative tumors. Pristauz et al. have reported that the expression of AR in the TNBC was higher in patients with BRCA1/2 mutations [30]. Gonzalez-Angulo et al. have recently evaluated the impact of the AR expression on outcome of 347 patients with breast cancer divided into three subsets: ER and PgR positive, HER2-positive, and TN. They demonstrated that high levels of AR were associated with a higher age at diagnosis, the expression of ER and PgR, and the presence of low nuclear grade. Significant differences in AR expression were observed between the different subtypes of breast cancer, in fact high expression levels of AR were observed in the ER and / or PgR positive one and low in the TN. At a median follow-up of 50 months the AR expression was an important prognostic factor for OS and DFS. In fact, high levels of AR correlated with a significantly reduced risk of relapse (HR 0:53, p = 0.002) and death (HR 0:57, p = 0.013) compared to low levels of expression. However, the subgroup analysis showed that in TNBC tumors expressing the androgen receptor there was a trend toward an increase in OS and RFS [29].

He et al. have retrospectively analyzed the prognostic value of AR in 287 patients TNBC treated at Sun Yat-sen University Cancer Center between January 1995 and December 2008. The AR expression was found in 74 patients (25.8%). This study demonstrated that the expression of AR is a favorable prognostic factor in terms of both OS (HR 0:34, p = 0.011) and DFS (HR 0:40, p = 0.008). In fact, both the DFS and the OS in patients were better in AR-positive (AR+) patients compared to those negative (87.0% vs. 74.2% and 94.2% vs 82.3%, respectively) [31]. Moreover, Yu et al. identified a 7-gene signature, including AR that may predict outcome among TNBC with known resistance to neoadjuvant chemotherapy. They confirmed the significantly positive correlation between AR expression and favorable survival in TNBC patients; indeed higher AR expression predicted a better relapse-free survival in patients with chemoresistant TNBC patients [45]. A recent systematic meta-analysis of 19 different studies showed that the expression of AR is favorable prognostic marker in early stage breast cancer, irrespective the ER status, with approximately a doubling of OS at 3 ad 5 years [46]. This growing interest on AR expression provided the rationale for the development of a phase II study with bicalutamide, a non-steroidal antiandrogen in the treatment of mTNBC—AR positive (NCT00468715). The study met its primary endpoint with an intriguing clinical benefit rate of 19% and a median PFS of 12 weeks among the 26 AR+ and ER/PgR- metastatic breast cancers with results comparable with single-agent or combination chemotherapy in other recent clinical trials conducted in TNBCs. However, given the aggressive course of TNBC, the presence of AR may identify a more indolent subtype of the disease, with peculiar clinical characteristics [47].

Another AR inhibitor, Enzalutamide, is currently been tested in a phase 2 study as a single agent in AR+ TNBC (NCT01889238).

Our study confirmed that lack of AR expression is a poor prognostic marker, since low AR expression correlated with a more aggressive tumor behavior and a poorer OS.

High Ki-67 expression has been shown to be associated with higher histologic grade, larger tumor size, positive lymph nodes status, short disease free-survival and overall survival in breast cancer [48, 49]. In addition, several studies showed a positive correlation between Ki67 expression and pathologic tumor response to neoadjuvant chemotherapy [4, 50]. In particular, Keam et al. have evaluated the prognostic and predictive role of Ki 67 in 105 TNBC patients with stage II or III treated with neoadjuvant chemotherapy based on docetaxel and doxorubicin, identifying two distinct subgroups of TN with different expression of Ki- 67, response and prognosis. The pCR was observed in 13.3% of patients. The subgroup with high Ki 67 (≥ 10%) reported a higher pCR compared with patients with low levels of Ki 67 [51]. Currently, there is no uniform consensus on a standardized cut-off value that might be used in the clinical practice, although staining levels of 10%–20% have been the most common to dichotomize populations used [52] and in the 13th St Gallen International Breast Cancer Conference (2013) a majority of the Panel voted that a threshold of ≥20% was indicative of ‘high’ Ki-67 status [42].

We confirmed that high Ki67 levels and the association with AR negative significantly correlated with a shorter OS and a more aggressive disease.

Although a loss of E-cadherin expression has been associated with larger tumor size, higher tumor grade, and lymph node metastasis in breast cancer [53, 54], reports in TNBC results remain limited and inconsistent.

Few studies have been reported so far evaluating the prognostic role of E-cadherin expression, either alone or in combination with other prognostic markers. Our report suggests that the lack of E-Cadherin expression is negatively associated with OS. Tang et al. evaluated the expression of AR and E-cadherin and their correlation with clinical and pathological characteristics of 127 patients TN. They found that highly undifferentiated tumors and menopausal status were associated with AR-negative status (p = 0.017) and positive lymph node status with loss of e-cadherin expression (p = 0.016). After a multivariate analysis tumor size, highly undifferentiated tumors, positive lymph node status, loss of AR and E-cadherin expression were significantly associated with DFS and the OS. In fact, patients with AR negative tumors had a worse outcome with a shorter DFS (p = 0.047) and OS (p = 0.038) as well as patients with E-cadherin negative expression showed a shorter DFS (p = 0.016) and OS (p = 0.012) [36]. Kashiwagi et al. have evaluated the prognostic role of E-cadherin and Ki-67 in 138 TN patients undergoing adjuvant chemotherapy. They showed that lack of expression of E-cadherin and the high expression of Ki-67 were associated with worse OS (p = <0.001). Moreover, a multivariate analysis of this study showed that the combination of E-cadherin negative and an elevated Ki 67 index was a strong predictor of poor overall survival in patients who received adjuvant chemotherapy, although they were not able to demonstrate a significant association between E-cadherin expression and clinic-pathological parameters [37].

## Conclusions

Based on current literature available, our data, albeit preliminary and based on a small retrospective series, confirmed the prognostic role of these molecular determinants and demonstrated that AR expression and high Ki-67 index levels are independent predictors of OS and are associated with a more aggressive clinico-pathological features, such as ductal histotype and with highly indifferentiated tumors. Our data suggest that the combination of AR and E-cadherin expression as well as Ki-67 status might be useful prognostic markers in TNBC. Hence, these molecular determinants could play an interesting role to classify subgroups of TNBC. Results of our study are intriguing and deserve further validation in large prospective studies before clinical implementation of these promising biomarkers in TNBC.

The role of several molecular determinants have been extensively evaluated in TNBC with contrasting results. Indeed, the clinical implementation of these biomarkers is yet to be ready since most of the studies reported so far contained largely non homogenous populations and analyzed only a limited panel of biomarkers, making difficult to draw any definitive conclusion. Our study is innovative since it is first proposed to simultaneously evaluate the role of most these molecular determinants. Our data suggest that the combination of AR and E-cadherin expression as well as Ki-67 status might be useful prognostic markers in TNBC in order to sub-classify the risk of these patients.
