# Correction: Androgen Receptor (AR), E-Cadherin, and Ki-67 as Emerging Targets and Novel Prognostic Markers in Triple-Negative Breast Cancer (TNBC) Patients

**DOI:** 10.1371/journal.pone.0132647

**Published:** 2015-07-02

**Authors:** Giuseppina Rosaria Rita Ricciardi, Barbara Adamo, Antonio Ieni, Luana Licata, Roberta Cardia, Giuseppa Ferraro, Tindara Franchina, Giovanni Tuccari, Vincenzo Adamo

There are a number of errors in the caption of Fig 1. Please see the complete, correct Fig 1 caption here.

**Fig 1:**
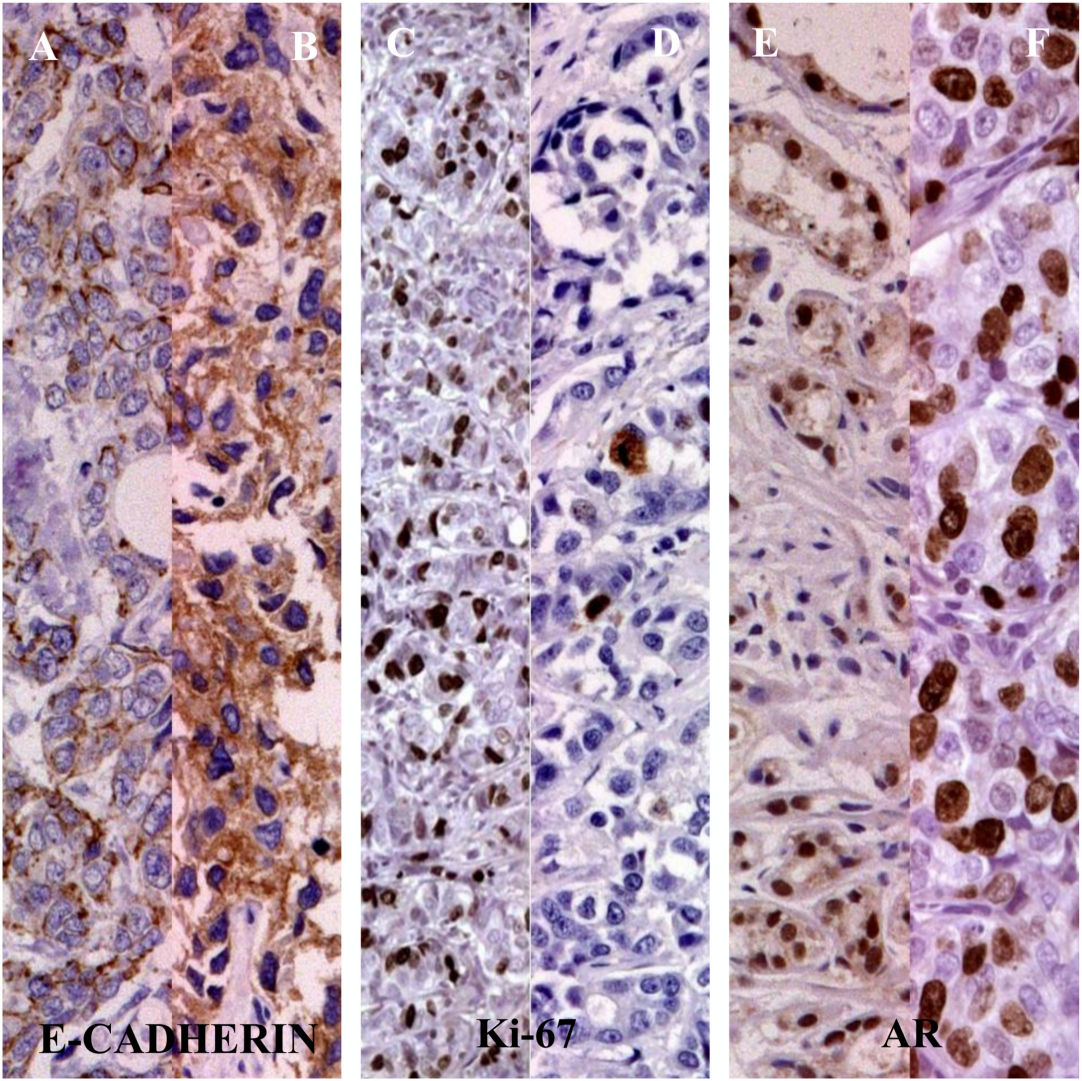
The E-cadherin, Ki-67 and AR expression in 45 patients TNBC. Legend: [A, B] E-cadherin negative/positive staining; [C,D] Ki-67 level < or ≥ 20%; [E,F] negative/positive AR staining.
